# Mitochondrial DNA mutations in preneoplastic lesions of the gastrointestinal tract: A biomarker for the early detection of cancer

**DOI:** 10.1186/1476-4598-5-73

**Published:** 2006-12-13

**Authors:** Guoping Sui, Shaoyu Zhou, Jean Wang, Marcia Canto, Edward E Lee, James R Eshleman, Elizabeth A Montgomery, David Sidransky, Joseph A Califano, Anirban Maitra

**Affiliations:** 1 Department of Pathology, Johns Hopkins University School of Medicine, Washington, DC; 2 Department of Otolaryngology and Head and Neck Surgery, Johns Hopkins University School of Medicine, Washington, DC; 3 Department of Medicine, Johns Hopkins University School of Medicine, Washington, DC; 4 Department of Oncology, Johns Hopkins University School of Medicine, Washington, DC; 5 McKusick-Nathans Institute of Genetic Medicine, Johns Hopkins University School of Medicine, Washington, DC; 6 Department of Pathology, Howard University School of Medicine, Washington, DC

## Abstract

**Background:**

Somatic mutations of mitochondrial DNA (mtDNA) are common in many human cancers. We have described an oligonucleotide microarray ("MitoChip") for rapid sequencing of the entire mitochondrial genome (Zhou et al, *J Mol Diagn *2006), facilitating the analysis of mtDNA mutations in preneoplastic lesions. We examined 14 precancerous lesions, including seven Barrett esophagus biopsies, with or without associated dysplasia; four colorectal adenomas; and three inflammatory colitis-associated dysplasia specimens. In all cases, matched normal tissues from the corresponding site were obtained as germline control. MitoChip analysis was performed on DNA obtained from cryostat-embedded specimens.

**Results:**

A total of 513,639 bases of mtDNA were sequenced in the 14 samples, with 490,224 bases (95.4%) bases assigned by the automated genotyping software. All preneoplastic lesions examined demonstrated at least one somatic mtDNA sequence alteration. Of the 100 somatic mtDNA alterations observed in the 14 cases, 27 were non-synonymous coding region mutations (i.e., resulting in an amino acid change), 36 were synonymous, and 37 involved non-coding mtDNA. Overall, somatic alterations most commonly involved the *COI*, *ND4 *and *ND5 *genes. Notably, somatic mtDNA alterations were observed in preneoplastic lesions of the gastrointestinal tract even in the absence of histopathologic evidence of dysplasia, suggesting that the mitochondrial genome is susceptible at the earliest stages of multistep cancer progression.

**Conclusion:**

Our findings further substantiate the rationale for exploring the mitochondrial genome as a biomarker for the early diagnosis of cancer, and confirm the utility of a high-throughput array-based platform for this purpose from a clinical applicability standpoint.

## Background

Somatic mutations of the mitochondrial DNA (mtDNA) are common in many human cancers, likely a reflection of altered DNA repair mechanisms and predisposition of mtDNA to free radical damage [1-3]. Although the functional significance of these mutations has been debated vis-à-vis their "cause and effect" relationship in cancer cells, there is little doubt that mtDNA mutations can serve an important role as a biomarker for human cancers [4-6]. Nevertheless, despite the frequency and widespread nature of mtDNA alterations in human cancers, the ability to use this property for biomarker development has been stymied due to the absence of high-throughput platforms for mutation detection. Unlike many nuclear genes that demonstrate mutational hotspots, mtDNA mutations can occur anywhere along the 16.5 kB bases of its genome [7-11]. Therefore, assays for mutational analysis of mtDNA in cancers, unlike those meant to detect specific metabolic disease-associated alterations, need to be "global". Even though certain focused assays such polymorphic repeat analysis of the so-called "D310 poly-C" tract in the non-coding D-loop have been designed [12-14], these, not unexpectedly, have lower sensitivities than what would be expected from complete sequencing of the mitochondrial genome. Moreover, mtDNA mutations in cancer are often heteroplasmic, and therefore, an added caveat over throughput and scale of the assay is its sensitivity for detecting aberrant clones within heterogenous templates [4].

In order to surmount these challenges of mtDNA mutational analysis in human cancers, we developed the first oligonucleotide-based sequencing microarray (MitoChip version 1.0) in 2004, which is capable of sequencing the mtDNA coding region on a single platform [15]. We and others have previously demonstrated the ability of this array for high throughput sequencing of the mtDNA genome, and confirmed the sensitivity and accuracy of this platform over conventional sequencing technologies [4,15-17]. More recently, we have validated a second generation oligonucleotide microarray (MitoChip version 2.0), that preserves many of the features of the earlier array, but has the added advantage of being able to sequence the entire 16,569 bases of mtDNA, including both the coding and non-coding regions [18]. The second generation arrays demonstrate >99.99% reproducibility of base calls in replicate experiments, and harbor greater sensitivity of detecting heteroplasmic mutations than either the first-generation MitoChip or conventional dye-terminator sequencing.

In this study, we utilize the MitoChip v2.0 arrays for detecting somatic mtDNA alterations in preneoplastic lesions of the gastrointestinal tract (Barrett esophagus, colorectal adenomas, and colitis-associated dysplasia). To our knowledge, this is the first report of complete mitochondrial genome sequencing in preneoplastic lesions of the GI tract, and provides unequivocal, albeit preliminary, evidence that mtDNA alterations are a frequent and early event in the multistep progression model of gastrointestinal neoplasia. These findings lay the groundwork for exploring mtDNA mutations as a biomarker for early detection of cancer utilizing high-throughput, array-based sequencing technologies.

## Materials and methods

### Tissue samples for mtDNA analysis

The studies performed were approved by the Institutional Review Boards of the Johns Hopkins University and Howard University School of Medicine. Fourteen individual preneoplastic lesions with matched normal samples, totaling 31 specimens overall, were accrued as follows (Table 1): seven Barrett esophagus cases were obtained from upper GI endoscopic biopsies, each from an individual patient. In all seven cases, normal squamous mucosa was obtained as a source of matched germline control. Cryosections were prepared from the snap-frozen biopsies to confirm histology of the lesions, performed by two trained gastrointestinal pathologists (A.M. and E.A.M.); of the cases utilized, two biopsies demonstrated Barrett mucosa without concomitant dysplasia, four exhibited low-grade dysplasia, and one demonstrated high-grade dysplasia, diagnosed using previously described histologic criteria for dysplasia in Barrett esophagus [19]. On the same lines, four colorectal adenomas were obtained from four individual patients, with biopsies of non-adenomatous colonic epithelium utilized as a source of germline control. Cryosections were prepared from snap-frozen excess adenoma tissue after samples had been submitted for routine histopathologic diagnosis, in order to confirm the diagnosis and integrity of tissue being submitted for sequencing array analysis. Of the four adenoma specimens, three were tubular adenomas and one was a sessile serrated adenoma (SSA) from the recto-sigmoid region [20,21]; none demonstrated evidence of invasive adenocarcinoma. Snap-frozen samples of colitis-associated dysplasia were obtained from three patients undergoing colectomy for long-standing idiopathic bowel disease (IBD) with concomitant dysplasia. Nine samples were obtained from the three colectomy specimens, and cryosections prepared in order to confirm the histopathologic diagnosis; three patients harbored dysplasia associated lesion or mass (DALM), a precursor of colitis-associated colorectal cancer [22,23]. Two patients had frank malignancies that were topographically distinct from the DALM lesions, and these were also submitted for array analysis. We were unable to obtain histologically unremarkable colonic mucosa in these patients, and therefore areas of inactive colitis was used as germline control, with the implicit understanding that these may potentially harbor mtDNA alterations distinct from true normal colonic mucosa; an area of active colitis was also examined in one patient. DNA was obtained from cryosections prepared from the 31 samples as described; no additional enrichment for a mitochondrial fraction is required for MitoChip analysis.

**Table 1:** Preneoplastic lesions of the GI tract used for mitochondrial DNA analysis

Case	Histology of Preneoplastic Lesion	Matched Normal
1	Barrett esophagus, negative for dysplasia	Yes; squamous mucosa
2	Barrett esophagus, negative for dysplasia	Yes; squamous mucosa
3	Barrett esophagus with low grade dysplasia	Yes; squamous mucosa
4	Barrett esophagus with low grade dysplasia	Yes; squamous mucosa
5	Barrett esophagus with low grade dysplasia	Yes; squamous mucosa
6	Barrett esophagus with low grade dysplasia	Yes; squamous mucosa
7	Barrett esophagus with high grade dysplasia	Yes; squamous mucosa
8	Tubular adenoma	Yes; colonic mucosa
9	Tubular adenoma	Yes; colonic mucosa
10	Tubular adenoma	Yes; colonic mucosa
11	Sessile serrated adenoma	Yes; colonic mucosa
12	Dysplasia associated lesion or mass (DALM)	No; inactive and active colitis
13	Dysplasia associated lesion or mass (DALM)	No; inactive colitis
14	Dysplasia associated lesion or mass (DALM)	No; inactive colitis

### PCR amplification, fragmentation, labeling and array hybridization

Samples were prepared for MitoChip analysis as we have previously described [18]. Briefly, long-range PCR was performed using three PCR primer sets that can amplify the entire mtDNA, using 100 ng of input DNA for each reaction. The cycling conditions for all reactions were: (1) 95°C for 2 min; (2) 95°C for 15 seconds; (3) 68°C for 7 min; (4) repeat step 2 for 29 times; (5) final extension for 12 min. As a control for PCR amplification and subsequent hybridization, a 7.5 kb plasmid DNA (Tag IQ-EX template) was amplified concomitantly with the test samples, using forward and reverse primers included in the CustomSeq™ kit (Affymetrix, Inc.). The procedures for sample pooling, DNA fragmentation and labeling are identical for both first and second-generation MitoChip assays, and a detailed protocol has been previously described [15,18]. Pre-hybridization, hybridization, washing, and scanning of the MitoChip were performed as described in the Affymetrix CustomSeq™ Resequencing protocol.

### Automated batch analysis of MitoChip data

The analysis of microarray data was performed using RA Tools, a modified version of the previously described adaptive background genotype-calling scheme (ABACUS) [24]; the open source software is available at [25]. Briefly, RA Tools uses an objective statistical framework to assign each genotype call a "quality score", which is the difference between the log (base 10) likelihood of the best fitting and the second best fitting statistical model for assigning a genotype at any position on the sequencing array. The total quality score threshold (totThresh) is the quality score that a given base has to exceed in order to be called. Increasing this value requires increased support for basecalls, and as a consequence, fewer bases are called. Bases that fail to reach this thresold are called "N." The optimum total threshold quality score was determined empirically to be 12, which yields the highest base call rate with the lowest discrepancy between genotypes for replicate samples, as previously described [18]. The Revised Cambridge Reference Sequence (RCRS) was used as the reference against which germline and lesional tissues were compared, and the MitoAnalyzer software [26] was used to query the impact on translated protein, if any, of mtDNA alterations.

### Microsatellite instability and identity analysis

Microsatellite instability analysis was performed on the one sessile serrated adenoma (SSA) specimen and its matched control tissue using the MSI Analysis System (Promega). In order to exclude the possibility of a sample mismatch, identity testing was performed on the SSA and its matched normal sample using the AmpF STR Identifiler PCR Amplification kit (Applied Biosystems).

## Results

**Figure 1:**
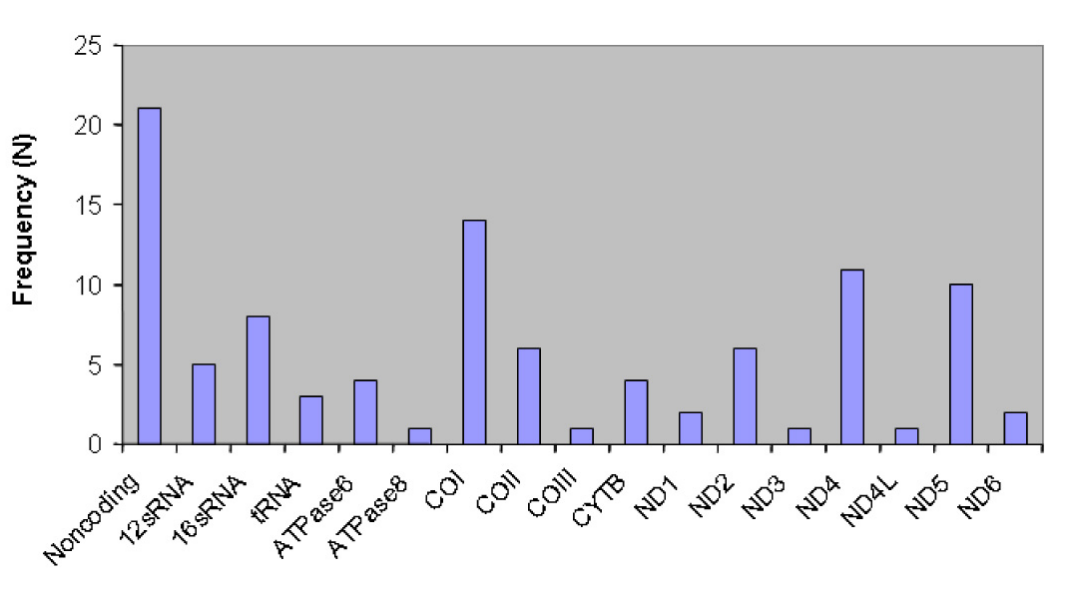
Distribution of somatic mtDNA alterations in preneoplastic lesions of the gastrointestinal tract by their location in the mitochondrial genome.

A total of 513,639 base pairs of mtDNA were sequenced across the 31 samples, at 16,569 bases per sample. Using the above described base calling criteria, 490,224 base pairs (95.4%) were assigned. The range of base calls was 88.2–96.9%, with a median call rate of 95.9% (Table 2). As detailed in Additional file 1, at least one somatic alteration in the mtDNA was identified in all 14 specimens of preneoplasia, with a range of 1–35 sequence variations compared to matched germline controls [see Additional file 1]. When stratified by their position in the mitochondrial genome, the sequence alterations were widely distributed, reiterating the need for a "global" sequencing strategy rather than focused mutational analysis. Of the 100 somatic sequence alterations, 27 (27%) were non-synonymous, i.e. resulting in an amino acid change, 36 (36%) were synonymous, and 37 (37%) involved the non-coding displacement D-loop, t-RNA and 12/16S RNA genes. As seen in Figure 1, the *COI*, *ND4 *and *ND5 *genes were the ones most commonly affected by sequence alterations, once the non-coding region was excluded. This distribution is comparable to the mutational spectrum reported in previous studies using cancer specimens [4,8-10].

When the individual samples and associated sequence alterations were studied, several notable trends emerged. The majority of sequence alterations were heteroplasmic in nature, which could either reflect emergence of an aberrant clone with intracellular heteroplasmy, or distinct homoplasmic cellular populations within the lesion, or both. Since samples were not microdissected and almost certainly contained non-lesional cell types (stroma, endothelium, etc.), it is likely that we are over-estimating the frequency of heteroplasmy. In the seven Barrett esophagus samples, we found no significant differences in the overall frequency of alterations between samples with no dysplasia and those with dysplastic epithelium; nevertheless, there was a trend towards higher rate of non-synonymous mutations in samples with dysplasia (9/23 or 39%) *versus *those without dysplasia (2/15 or 13%). With the exception of a recurrent sequence alteration in 16S RNA at nucleotide position 1949 in two Barrett samples, other somatic variations were unique to each specimen.

**Figure 2:**
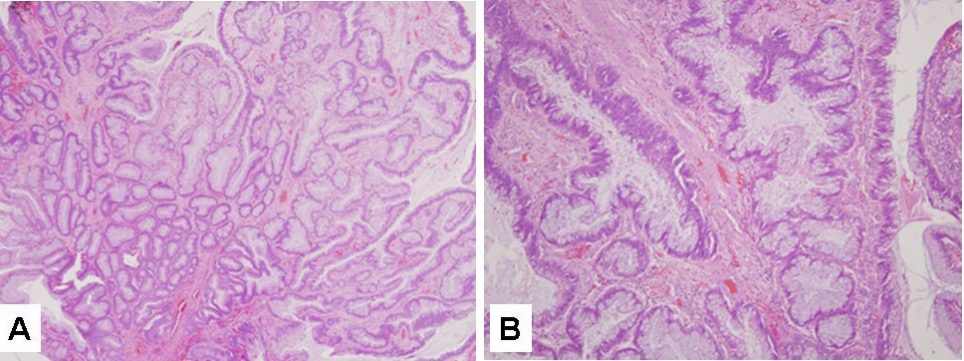
Sessile serrated adenoma with a mitochondrial mutator phenotype. (A) Low power and (B) high power demonstrating the serrated architecture of the glands. Hematoxylin and eosin stain.

Several recurrent (i.e., in more than one specimen) sequence alterations were seen in the four adenoma samples [see Additional file 1], but one non-synonymous mutation in particular, Thr415Ala in the *COI *gene was observed in 3 of 4 adenomas, suggesting a putative pathogenic role. Of the four colorectal polyps, one specimen (a recto-sigmoid sessile serrated adenoma [SSA], Figure 2) had 35 mtDNA alterations, including six non-synonymous mutations. Notably, all but one of the sequence alterations was a transition. In order to exclude the possibility of sample mismatch, we performed identity testing on DNA from the normal colonic mucosa and the SSA, and this analysis confirmed a perfect match. Nuclear microsatellite instability (MSI) was excluded by analysis of matched normal and adenoma DNA; i.e., per the Bethesda criteria [27], the SSA was "microsatellite stable" (*data not shown*). Three of 3 colitis-associated dysplasia samples demonstrated at least one somatic alteration, with the associated cancers demonstrating additional mtDNA changes. Of note, the two associated carcinomas shared a subset of genetic alterations with the synchronous, albeit topographically distinct, dysplastic lesions, arguing for a potential field defect shared across the colonic epithelium. However, no shared alterations were seen across the three IBD cases.

## Discussion

Array-based analysis of the entire mitochondrial genome demonstrates that precursor lesions of epithelial cancers harbor somatic mtDNA alterations much like their invasive counterparts. Limited sequencing of mtDNA using conventional techniques has previously identified somatic alterations in precursor lesions. For example, a poly-C tract known as D310 in the non-coding D-loop is commonly mutated in many cancers, and was recently been shown to be mutated in precursor lesions of head and neck cancer, esophageal adenocarcinoma, and gallbladder adenocarcinoma [13,28,29]. The current study provides, for the first time, an insight into the prevalence and distribution of mtDNA alterations in precursor lesions on a "global" scale. The rationale for preferential sequencing of the non-coding region of mtDNA has been the assumption that this portion of the mitochondrial genome is particularly susceptible to the rigors of DNA damage encountered in metabolically active cancer cells [30]. However, our studies and those of others continue to provide evidence that the coding region is at least equally susceptible to DNA alterations in cancer [4,7]. In as many as 7 of 14 (50%) preneoplastic samples, we detected somatic alterations restricted to the coding region of mtDNA, while two additional cases had only single non-coding D-loop changes in addition to coding region alterations [see Additional file 1]. In addition, the relatively low level of recurrent mutations at any given nucleotide position also suggests that selective sequencing of so-called "hot spots" may not provide the same yield as sequencing analogous regions in nuclear genes (for example, the *KRAS *codon 12 or the *BRAF *V599 mutations). Both of theses caveats have enormous implications in terms of sensitivity in the context of cancer biomarker studies, and underscore the importance of complete mtDNA sequencing.

In our series, we were able to detect at least one somatic alteration in all preneoplastic samples analyzed, although there was a wide range (1 – 35) in the number of variants within a given case. It is important to stress that chip-based chemistry is best suited for detection of single base pair alterations [24,31], and it is likely that we are missing insertions/deletions, frameshift mutations, and large deletions, all of which have been reported in cancer samples. In addition, because we are not using lymphocyte DNA as a germline control, there is a potential for underestimating the true frequency of somatic alterations, since some mtDNA alterations will be common in both histologically "normal" tissue and preneoplasia, as a result of a field defect. Nevertheless, there does not appear to be a significant attrition in terms of false negative cases, as we are able to demonstrate at least one somatic alteration in all 14 of preneoplastic lesions examined, when compared to the paired histologically normal specimen.

Barrett esophagus is the recognized precursor lesion of esophageal adenocarcinoma, and the prevalence of this disease is dramatically increasing in the Caucasian population [32]. Patients with Barrett's esophagus have a 30- to 125-fold increased risk of the development of esophageal cancer in comparison with the general population. Unfortunately, the cancer usually manifests at an advanced stage, such that esophageal adenocarcinoma carries a high mortality rate [33]. The American Cancer Society estimates that there will be about 14,550 new cases of this cancer in 2006, and about 13,770 people will die of the disease. Thus, there is an urgent need to develop effective early detection strategies for Barrett esophagus-associated adenocarcinomas, prior to the onset of invasive cancer. Our studies confirm that somatic mtDNA alterations are a frequent accompaniment of the metaplastic epithelium characteristic of Barrett esophagus. Of note, we detect mtDNA alterations even at the earliest histologic stages of the multistep progression model of esophageal adenocarcinoma, namely, in Barrett mucosa without evidence of dysplasia, and find no significant differences in the overall frequency of sequence variations between Barrett mucosa with or without dysplasia. On the contrary, there is a trend towards higher rate of non-synonymous mutations in samples with dysplasia (9/23 or 39%) *versus *those without dysplasia (2/15 or 13%). The significance of this latter difference is uncertain and will need to be confirmed in larger series; however, one can speculate that the increased prevalence of non-synonymous changes, with the potential effect on mitochondrial protein function, might provide a selective advantage in the advanced histologic lesions. The presence of clonal genetic alterations in non-dysplastic Barrett epithelium is not unexpected; for example, we have reported the occurrence of homozygous chromosome 9p21 deletions, encompassing the *CDKN2A/p16 *gene, in 16% of Barrett epithelia even in the absence of dysplasia [34]. Meltzer and colleagues recently performed a global expression analysis of Barrett esophagus, normal squamous mucosa and esophageal adenocarcinoma, and found that molecular events at the transcriptional level in Barrett esophagus are remarkably similar to Barrett-associated adenocarcinoma [35]. Therefore, despite the "innocuous" histology, Barrett mucosa, even when non-dysplastic, may be more ominous than previously considered at a molecular level.

There are two major pathways to colorectal carcinogenesis – one preceded by the development of adenomas, and the other in the backdrop of long-standing inflammatory colitis, including ulcerative colitis and Crohn's disease [36,37]. Despite the eventual progression to colorectal adenocarcinomas, the genetic mechanisms implicated are quite distinct in the two pathways. For example, the adenoma-carcinoma sequence is characterized by nearly ubiquitous aberrations in the *wnt *signaling pathway, such as *APC *or *β-catenin *gene mutations [38]. In contrast, colitis-associated dysplasia and carcinomas commonly demonstrate abnormalities of p53 gene function as an early event [39]. We examined colorectal cancer precursor lesions from both scenarios (adenomas and IBD dysplasia), and identified mtDNA alterations in all seven specimens. Of note, we detected multiple somatic alterations that were present in more than 1 adenoma; in particular a Thr415Ala mutation in the *COI *gene (RCRS position 7146) was present in 3 of 4 adenomas. Curiously, this identical mutation has been previously reported in association with Alzheimer disease [40], but we could not determine a reported association with cancer. Nevertheless, its occurrence in multiple adenomas suggests a putative pathogenic role, as well as the potential for its exploitation as a cancer biomarker in clinical samples, both of which contentions need to be borne out in further studies using an expanded sample of adenomas. Mutations of the cytochrome oxidase gene family with corresponding biochemical defects have been reported not only in colorectal carcinomas, but also in isolated colonic crypts [41,42]. In fact, it has been hypothesized that these early genetic lesions may "mark" the colonic crypt cells eventually progressing to cancer, although their precise contributory role to neoplastic progression is of course difficult to determine. The absence of the Thr415Ala mutation, or other adenoma-associated sequence alterations, in the three colitis-associated dysplasia cases also reiterates what is known from the study of nuclear genes; i.e., the genetic pathways in the two precursor lesions of colorectal cancer are distinct. However, one needs to stress the relatively few numbers of cases in each category, and larger numbers of cases may cement this contention.

In our series, we also had the opportunity to examine a sessile serrated adenoma (SSA), which harbored a striking number (N = 35) of somatic mtDNA alterations, all but one of which were transition mutations. We were able to exclude a sample mismatch by identity testing, which may have been a potential explanation for the sequence discrepancies. Given the established association between SSAs and *nuclear *mismatch repair (MMR) deficiency [21,43,44], which likely accounts for the increased predisposition of these lesions to progress to invasive adenocarcinomas, we assessed MMR status in the SSA. Based on absence of the Bethesda criteria for MMR deficiency, we confirmed that nuclear DNA from the SSA was microsatellite stable. However, MMR is only one of the six major types of DNA repair existent in the cell, and most pathways have distinct nuclear and mitochondrial components [45,46]. The underlying basis for the exceptionally large number of mtDNA alterations in this "mitochondrial mutator" adenoma remains unknown, and a larger series of colorectal adenomas of the serrated and non-serrated subtypes would have to be examined in order to determine whether our rudimentary observation in a single SSA bears out in other specimens of this nature.

In summary, our preliminary study confirms that mtDNA alterations are highly prevalent in preneoplastic lesions of the gastrointestinal tract. This is the first study to demonstrate feasibility of using an array-based approach for complete mitochondrial sequencing of preneoplastic lesions, and adds to the cumulative evidence that mtDNA is a rational biomarker for the early detection of cancer.

**Table 2A:** Summary of array-based analysis for mtDNA alterations

Number of samples analyzed =	31
MtDNA bases per MitoChip v2.0 microarray =	16,569
Total number of mtDNA bases sequenced =	513,639
Total number of mtDNA bases assigned by genotyping software =	490,224
Percent overall base call rate =	95.4%
Range of bases called across 31 arrays =	88.2–96.9%
Median base call rate =	95.9%

**Table 2B:** MitoChip base calls in the individual lesions and matched normal samples (denominator 16,569 bases in all cases)

	Normal	Barrett		
Case 1	15789	15992		
Case 2	15868	15858		
Case 3	16017	15886		
Case 4	15444	15988		
Case 5	15904	15983		
Case 6	15649	15770		
Case 7	15672	15483		
				
	Normal	Adenoma		

Case 8	15954	15755		
Case 9	15619	15814		
Case 10	14609	15741		
Case 11	15976	15787		
				
	Inactive	Active	DALM	Cancer

Case 12	15665	15985	15964	16010
Case 13	16025		16014	
Case 14	15920		16028	16055

## Supplementary Material

Additional file 1 **MtDNA sequence alterations across 14 preneoplastic lesions of the gastrointestinal tract**. The table enumerates the mitochondrial DNA sequence alterations detected in the individual preneoplastic lesions, and their effect on the translated protein, if any. Click here for file
