# Supplementary material for: Mitochondrial DNA mutations in preneoplastic lesions of the gastrointestinal tract: A biomarker for the early detection of cancer
Source: Mol Cancer. 2006 Dec 13;5:73. doi: 10.1186/1476-4598-5-73 (PMC1764424; doi:10.1186/1476-4598-5-73)
Supplement: Additional file 1 — MtDNA sequence alterations across 14 preneoplastic lesions of the gastrointestinal tract. The table enumerates the mitochondrial DNA sequence alterations detected in the individual preneoplastic lesions, and their effect on the translated protein, if any. [file 1476-4598-5-73-S1.doc]

**MtDNA sequence alterations across 14 preneoplastic lesions of the gastrointestinal tract**

| ***Case 1*** | *RCRS Position* | *RCRS Sequence* | *Normal* | *Barrett, No dysplasia* |  |  | *Gene* | *Amino Acid* | *Non-synonymous* |
| --- | --- | --- | --- | --- | --- | --- | --- | --- | --- |
|  | 73 | A | G | G+A |  |  | Non coding | NA | NA |
|  | 131 | T | T | T+C |  |  | Non coding | NA | NA |
|  | 5401 | T | T | T+C |  |  | ND2 | Val311Ala | Yes |
|  | 13155 | C | C | C+T |  |  | ND5 | Ile273 | No |
|  | 14905 | G | G | G+A |  |  | CYTB | Met53 | No |
|  | 16311 | T | T | T+C |  |  | Non coding | NA | NA |
|  | 16327 | C | C | C+T |  |  | Non coding | NA | NA |
|  |  |  |  |  |  |  |  |  |  |
| ***Case 2*** | *RCRS Position* | *RCRS Sequence* | *Normal* | *Barrett, No dysplasia* |  |  | *Gene* | *Amino Acid* | *Non-synonymous* |
|  | 199 | T | C | C+T |  |  | Non coding | NA | NA |
|  | 1719 | G | A | A+G |  |  | 16sRNA | NA | NA |
|  | 10873 | T | T | T+C |  |  | ND4 | Pro38 | No |
|  | 12501 | G | A | A+G |  |  | ND5 | Met55 | No |
|  | 14318 | T | T | T+C |  |  | ND6 | Asn119Ser | Yes |
|  | 14783 | T | T | T+C |  |  | CYTB | Leu13 | No |
|  | 15924 | A | G | G+A |  |  | tRNA | NA | NA |
|  | 16298 | T | T | T+C |  |  | Non coding | NA | NA |
|  |  |  |  |  |  |  |  |  |  |
| ***Case 3*** | *RCRS Position* | *RCRS Sequence* | *Normal* | *Low Grade Dysplasia* |  |  | *Gene* | *Amino Acid* | *Non-synonymous* |
|  | 185 | G | G | G+A |  |  | Non coding | NA | NA |
|  | 228 | G | G | G+A |  |  | Non coding | NA | NA |
|  | 295 | C | C | C+T |  |  | Non coding | NA | NA |
|  | 489 | C | T | T+C |  |  | Non coding | NA | NA |
| ****** | **1949** | G | G | G+C |  |  | 16sRNA | NA | NA |
|  | 2387 | T | T | T+C |  |  | 16sRNA | NA | NA |
|  | 4216 | T | T | T+C |  |  | ND1 | Tyr304His | Yes |
|  | 5198 | A | A | A+G |  |  | ND2 | Leu243 | No |
|  | 8994 | G | A | A+G |  |  | ATPase6 | Leu156 | No |
|  | 10398 | A | A | A+G |  |  | ND3 | Thr114Ala | Yes |
|  |  |  |  |  |  |  |  |  |  |
| ***Case 4*** | *RCRS Position* | *RCRS Sequence* | *Normal* | *Low Grade Dysplasia* |  |  | *Gene* | *Amino Acid* | *Non-synonymous* |
|  | 11106 | T | T | T+C |  |  | ND4 | Ile116Thre | Yes |
|  |  |  |  |  |  |  |  |  |  |
| ***Case 5*** | *RCRS Position* | *RCRS Sequence* | *Normal* | *Low Grade Dysplasia* |  |  | *Gene* | *Amino Acid* | *Non-synonymous* |
| ****** | **1949** | G | G | G+C |  |  | 16sRNA | NA | NA |
|  | 4752 | T | T | C+T |  |  | ND2 |  | Yes |
|  |  |  |  |  |  |  |  |  |  |
| ***Case 6*** | *RCRS Position* | *RCRS Sequence* | *Normal* | *Low Grade Dysplasia* |  |  | *Gene* | *Amino Acid* | *Non-synonymous* |
|  | 795 | A | A | A+C |  |  | 12sRNA | NA | NA |
|  | 1103 | A | A | A+G |  |  | 12sRNA | NA | NA |
|  | 1414 | C | C | C+G |  |  | 12sRNA | NA | NA |
|  | 4519 | C | C | C+A |  |  | ND2 | Thr17Lys | Yes |
|  | 5968 | T | T | T+C |  |  | COI | Phe22Ser | Yes |
|  |  |  |  |  |  |  |  |  |  |
| ***Case 7*** | *RCRS Position* | *RCRS Sequence* | *Normal* | *High Grade Dysplasia* |  |  | *Gene* | *Amino Acid* | *Non-synonymous* |
|  | 5628 | T | T | T+C |  |  | tRNA | NA | NA |
|  | 5950 | G | G | G+A |  |  | COI | Gly16Glu | Yes |
|  | 10901 | A | A | A+G |  |  | ND4 | Asn48Asp | Yes |
|  | 11324 | T | T | T+G |  |  | ND4 | Ser189Ala | Yes |
|  | 16179 | C | C | C+T |  |  | Non coding | NA | NA |
|  |  |  |  |  |  |  |  |  |  |
| ***Case 8*** | *RCRS Position* | *RCRS Sequence* | *Normal* | *Tubular Adenoma* |  |  | *Gene* | *Amino Acid* | *Non-synonymous* |
|  | 912 | T | T | T+A |  |  | 12s RNA | NA | NA |
|  |  |  |  |  |  |  |  |  |  |
| ***Case 9*** | *RCRS Position* | *RCRS Sequence* | *Normal* | *Tubular Adenoma* |  |  | *Gene* | *Amino Acid* | *Non-synonymous* |
|  | 680 | T | C | C+T |  |  | 12sRNA | NA | NA |
| ****** | **1738** | T | T | T+C |  |  | 16sRNA | NA | NA |
|  | 2885 | T | T | T+C |  |  | 16sRNA | NA | NA |
|  | 5393 | T | T | T+C |  |  | ND2 | Ser308 | No |
|  | 5655 | T | T | T+C |  |  | tRNA | NA | NA |
| ******* | **7146** | A | A | A+G |  |  | COI | Thr415Ala | Yes |
| ****** | **7867** | C | C | C+T |  |  | COII | Ser94 | No |
| ****** | **16093** | T | C | C+T |  |  | Non coding | NA | NA |
|  |  |  |  |  |  |  |  |  |  |
| ***Case 10*** | *RCRS Position* | *RCRS Sequence* | *Normal* | *Tubular Adenoma* |  |  | *Gene* | *Amino Acid* | *Non-synonymous* |
| ****** | **1738** | T | T | T+C |  |  | 16sRNA | NA | NA |
| ******* | **7146** | A | A | A+G |  |  | COI | Thr415Ala | Yes |
|  | 7173 | A | A | A+C |  |  | COI | Thr424Pro | Yes |
|  | 7274 | C | T | T+C |  |  | COI | Gly457 | No |
|  | 7771 | A | G | G+A |  |  | COII | Glu62 | No |
| ****** | **7867** | C | C | C+T |  |  | COII | Ser94 | No |
| ****** | **8248** | A | A | A+G |  |  | COII | Met221 | No |
| ****** | **10810** | T | T | T+C |  |  | ND4 | Leu17 | No |
|  | 11046 | T | T | T+C |  |  | ND4 | Leu96Pro | Yes |
|  |  |  |  |  |  |  |  |  |  |
| ***Case 11*** | *RCRS Position* | *RCRS Sequence* | *Normal* | *Sessile serrated adenoma* |  |  | *Gene* | *Amino Acid* | *Non-synonymous* |
|  | 146 | T | T | T+C |  |  | Non coding | NA | NA |
|  | 204 | T | T | T+C |  |  | Non coding | NA | NA |
|  | 6548 | C | C | T |  |  | COI | Leu215 | No |
|  | 6827 | T | T | C |  |  | COI | Ala308 | No |
|  | 6989 | A | A | G |  |  | COI | Ser362 | No |
|  | 7055 | A | A | G |  |  | COI | Gly384 | No |
| ******* | **7146** | A | A | G |  |  | COI | Thr415Ala | Yes |
|  | 7389 | T | T | C |  |  | COI | Tyr496His | Yes |
|  | 7915 | C | C | T |  |  | COII | Tyr110 | No |
| ****** | **8248** | A | A | G |  |  | COII | Met221 | No |
|  | 8468 | C | C | T |  |  | ATPase8 | Leu35 | No |
|  | 8655 | C | C | T |  |  | ATPase6 | Ile43 | No |
|  | 8772 | T | C | T |  |  | ATPase6 | Thr82 | No |
|  | 9063 | A | G | A |  |  | ATPase6 | Leu179 | No |
|  | 9221 | A | G | A |  |  | COIII | Ser5 | No |
|  | 10522 | G | G | G+A |  |  | ND4L | Gly18Glu | Yes |
|  | 10790 | T | C | T |  |  | ND4 | Leu11 | No |
| ****** | **10810** | T | T | C |  |  | ND4 | Leu17 | No |
|  | 11709 | T | T | T+C |  |  | ND4 | Ile317Thre | Yes |
|  | 11914 | G | G | G+A |  |  | ND4 | Thr385 | No |
|  | 11944 | T | T | T+C |  |  | ND4 | Leu395 | No |
|  | 12519 | T | C | C+T |  |  | ND5 | Val61 | No |
|  | 12693 | A | A | A+G |  |  | ND5 | Lys119 | No |
|  | 13506 | C | T | T+C |  |  | ND5 | Tyr390 | No |
|  | 13590 | G | G | G+A |  |  | ND5 | Leu418 | No |
|  | 13803 | A | A | A+G |  |  | ND5 | Thr489 | No |
|  | 13958 | G | G | G+C |  |  | ND5 | Gly541Ala | Yes |
|  | 14203 | A | G | G+A |  |  | ND6 | Gly157 | No |
|  | 15301 | G | G | G+A |  |  | CYTB | Leu185 | No |
|  | 15849 | C | C | C+T |  |  | CYTB | Thr368Ile | Yes |
| ****** | **16093** | T | T | T+C |  |  | Non coding | NA | NA |
|  | 16126 | T | C | C+T |  |  | Non coding | NA | NA |
|  | 16264 | C | T | C |  |  | Non coding | NA | NA |
|  | 16270 | C | C | T |  |  | Non coding | NA | NA |
|  | 16519 | T | C | C+T |  |  | Non coding | NA | NA |
|  |  |  |  |  |  |  |  |  |  |
| ***Case 12*** | *RCRS Position* | *RCRS Sequence* | *Inactive Colitis* | *Active Colitis* | *DALM* | *Cancer* | *Gene* | *Amino Acid* | *Non-synonymous* |
|  | 3736 | G | G | G | G+A | G+A | ND1 | Val144Ile | Yes |
|  | 6208 | T | T | T | T | T+C | COI | Phe102Ser | Yes |
|  | 4794 | G | G | G | G+A | G+A | ND2 | Ala109Thr | Yes |
|  |  |  |  |  |  |  |  |  |  |
| ***Case 13*** | *RCRS Position* | *RCRS Sequence* | *Inactive Colitis* | *DALM* |  |  | *Gene* | *Amino Acid* | *Non-synonymous* |
|  | 456 | C | C | C+T |  |  | Non coding | NA | NA |
|  | 1816 | G | G | G+A |  |  | Non coding | NA | NA |
|  | 7440 | T | T | T+C |  |  | COI | Ser513Pro | Yes |
|  |  |  |  |  |  |  |  |  |  |
| ***Case 14*** | *RCRS Position* | *RCRS Sequence* | *Inactive Colitis* | *DALM* | *Cancer* |  | *Gene* | *Amino Acid* | *Non-synonymous* |
|  | 2680 | T | T | T | T+C |  | 16sRNA | NA | NA |
|  | 12611 | T | T | T | T+C |  | ND5 | Val92Ala | Yes |
|  | 13676 | A | A | A+G | A+G |  | ND5 | Asn447Ser | Yes |

**Note**: Cases are numbered as designated in Table 1. RCRS = Revised Cambridge Reference Sequence; both the RCRS position and the reference nucleotide at that position are designated. “Normal” refers to the actual nucleotide present in non-neoplastic tissues for that individual, and may or may not concur with the expected RCRS reference sequence. The MitoAnalyzer tool (<http://www.cstl.nist.gov/biotech/strbase/mitoanalyzer.html>) was used for determining the effect of base substitution on translated protein sequence. Base positions highlighted in yellow are recurrent alterations present in >1 sample, while base positions highlighted in purple are present in >2 samples.
